# The *mir-35-42* binding site in the *nhl-2* 3’UTR is dispensable for development and fecundity

**DOI:** 10.17912/micropub.biology.000241

**Published:** 2020-04-14

**Authors:** Bing Yang, Katherine McJunkin

**Affiliations:** 1 Laboratory of Cellular and Developmental Biology, NIDDK Intramural Research Program, Bethesda, MD 20892

**Figure 1:**
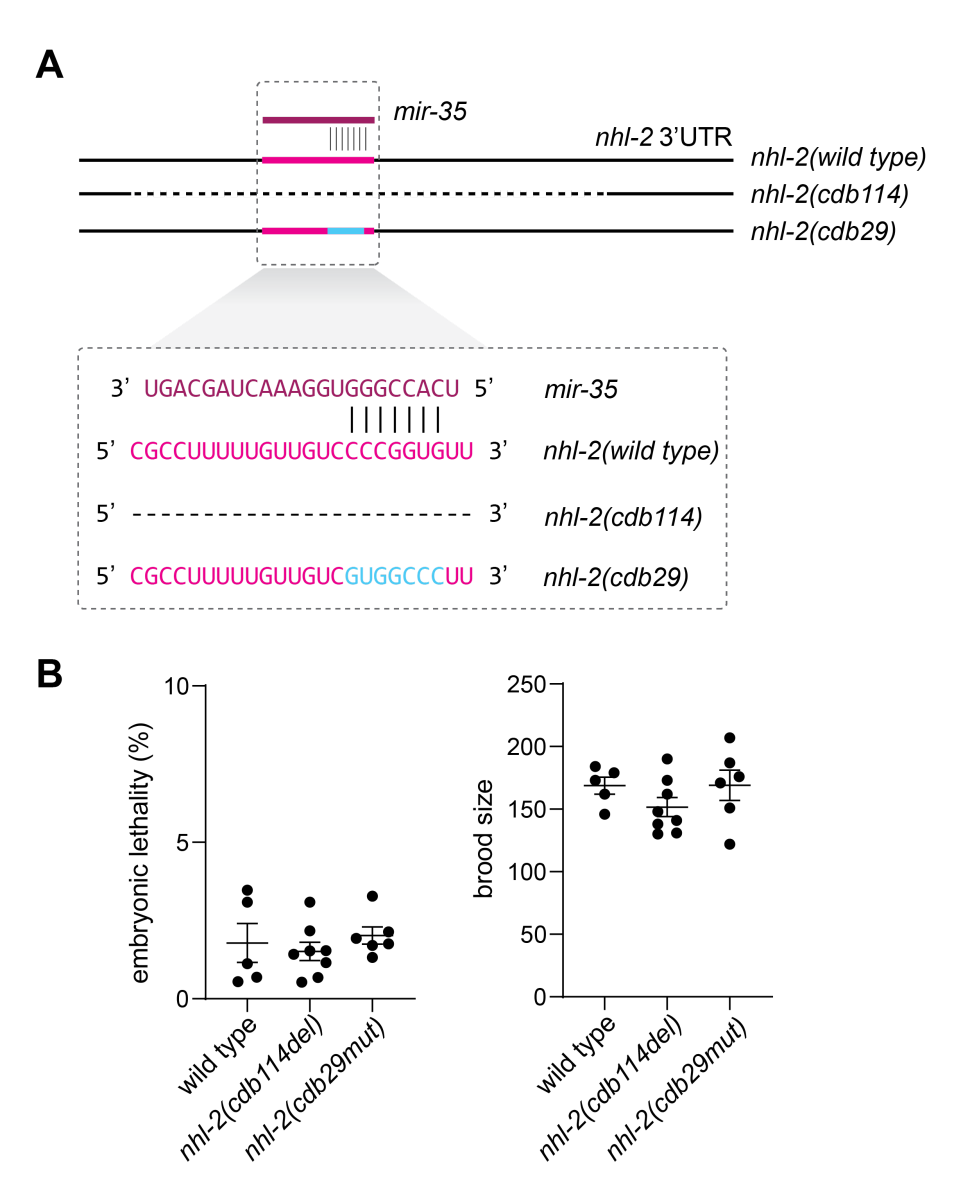
**CRISPR mutations demonstrate that the *mir-35-42* binding site in *nhl-2* is not essential for development or fecundity.** (A) Schematics depicting the region of the *nhl-2* 3’UTR containing two mutations affecting the *mir-35-42* binding site. *nhl-2(cdb114)* is a 75-bp deletion encompassing the binding site, whereas *nhl-2(cdb29)* is a mutation that reverses the sequence of the seed-binding site, thus abolishing predicted base pairing to *mir-35* (or its family members *mir-36-42*). (B) Quantification of embryonic lethality and brood size in homozygous mutant *nhl-2* lines as shown in (A).

## Description

The *mir-35­-42* family of microRNAs (miRNAs) acts redundantly to ensure embryonic viability in *C. elegans* (Alvarez-Saavedra and Horvitz 2010). We are interested in defining the essential targets that must be repressed by the *mir-35-42* family. Our previous work suggested that *NHL (ring finger b-box coiled coil) domain containing 2 (nhl-2)* may be one such target because genome editing attempts to delete the *mir-35-42* seed binding region in the *nhl-2* 3’UTR were unsuccessful (McJunkin and Ambros 2017). The same CRISPR reagents were successful at creating such a deletion in a background containing an NHL-2 CDS deletion (*nhl-2(ok818)*) (McJunkin and Ambros 2017). Together, we took these results to mean that derepression of *nhl-2* induced lethality or sterility, preventing our isolation of the deletion lines in the wild type context. More recently, CRISPR genome editing reagents and protocols have become many-fold more efficient, most notably by injection of recombinant Cas9 RNPs pre-loaded with synthetic guide RNAs (gRNAs) (Paix *et al.* 2014). Using injection of Cas9/gRNA RNPs, we have succeeded in deleting and mutating the *mir-35-42* seed binding region in the *nhl-2* 3’UTR in a wild type background (see alleles in Figure 1A). Because such alleles were previously difficult to generate, we quantified their fecundity and embryonic viability (which are two aspects of physiology affected by *mir-35* family mutations) (Alvarez-Saavedra and Horvitz 2010; McJunkin and Ambros 2014) to see if they were impaired, but we found these animals to be wild type (Figure 1B). Therefore, our original interpretation – that the difference in CRISPR editing between wild type and *nhl-2(ok818)* backgrounds was due to negative selection of miRNA binding site mutations in the wild type background – was incorrect. One possible explanation for the observed difference in editing may be alterations in chromatin structure induced by the 1.5kb *nhl-2(ok818)* deletion. Indeed, nucleosome position and dynamics have been shown to alter efficiency of Cas9 cleavage (Chen *et al.* 2016; Horlbeck *et al.* 2016; Isaac *et al.* 2016; Hinz *et al.* 2016; Daer *et al.* 2017; Yarrington *et al.* 2018; Kim and Kim 2018). Thus, differences in genome editing efficiencies between genetic backgrounds should be interpreted with caution.

## Methods

N2 adult hermaphrodites were injected with Cas9/gRNA RNPs to perform CRISPR. For *nhl-2(cdb29)*, the injection mix contained 6μM homemade Cas9, 1.4μM each of three gRNAs (gKM1, gKM20, and gKM3), 27ng/μl of *dpy-10* ssDNA oligo repair donor, and 164ng/μl of *nhl-2* ssDNA oligo repair donor (gKM102) (Paix *et al.* 2014; Arribere *et al.* 2014). The injection mix for *cdb114* contained 2μM IDT Cas9, 1μM of gKM26, and 1μM of gKM3. F1 animals with Dpy or Rol phenotype indicating co-CRISPR at *dpy-10* were isolated and genotyped by PCR. Genotyping primers are oKM85 and oKM86, which yield a 331-bp fragment in wild type or *cdb29* and 256-bp fragment in *cdb114*. Wild type and *cdb29* fragments are distinguished by digestion with NciI, which cuts the wild type PCR product into two fragments (87-bp and 224-bp). All guides were AltR crRNAs from IDT preannealed with IDT tracrRNA, except for gKM20 which was a Synthego sgRNA. Strains were homozygosed and segregated away from *dpy-10* mutations (not further backcrossed) and scored for fecundity and viability at 25°C.

The protospacer sequences used:

gKM1 ATCCGCCTTTTTGTTGTCCC

gKM3 GCTACCATAGGCACCACGAG – dpy-10 protospacer from (Arribere *et al.* 2014)

gKM20 AAAATAATGGAACAACACCG

gKM26 GATGACGGAACGGTGTCACC

Oligonucleotide sequences:

oKM85 GGTCACATTGTGACGTTGTGTAAG

oKM86 GTGGCAAATGAGGTCTCAAAACG

oKM102

CCGTCTTCTTTTTTTTCCTTCTCCCTTTGCTTATCCGCCTTTTTGTTGTC**GTGGCCC**TTGTTCCATTATTTTAAGTTCCTAAGTTTCTTTCCCCTCCCCA

*dpy-10* repair donor

CACTTGAACTTCAATACGGCAAGATGAGAATGACTGGAAACCGTACCGCATGCGGTGCCTATGGTAGCGGAGCTTCACATGGCTTCAGACCAACAGCCTAT

## Reagents

MCJ71 *nhl-2(cdb29)* III

MCJ236 *nhl-2(cdb114)* III

[The *cdb114* breakpoints are as follows: TCCTTCTCCCTTTGCTTATC—75bp deletion—TTCTTTCGTTTTGAGACCTC]
